# Renin‐Angiotensin and Endothelin Systems in Patients Post‐Takotsubo Cardiomyopathy

**DOI:** 10.1161/JAHA.122.025989

**Published:** 2022-07-13

**Authors:** Hilal Khan, Amelia Rudd, David T. Gamble, Alice M. Mezincescu, Lesley Cheyne, Graham Horgan, Neeraj Dhaun, David E. Newby, Dana K Dawson

**Affiliations:** ^1^ University of Aberdeen United Kingdom; ^2^ University of Edinburgh United Kingdom

**Keywords:** angiotensin, cardiomyopathy, endothelin, renin, takotsubo, ACE/Angiotension Receptors/Renin Angiotensin System, Biomarkers, Myocardial Biology, Cardiomyopathy, Heart Failure

## Abstract

**Background:**

We investigate if renin‐angiotensin and endothelin‐1 response pathways follow the same pattern of recovery as left ventricular ejection fraction in patients with takotsubo cardiomyopathy.

**Methods and Results:**

Ninety patients with takotsubo cardiomyopathy (n=30 in each of “acute,” “convalescent” [3–5 months] and “recovered” [>1 year] groups) who were on minimal or no medication and were free of any significant cardiac/metabolic comorbidities, and 30 controls were studied. Serum concentrations of renin, angiotensin‐converting enzyme, angiotensin II, big endothelin‐1, endothelin‐1 were measured using commercially available ELISA, and B‐type natriuretic peptide was measured using an immunoassay. Mean left ventricular ejection fraction was <40% during the acute phase in all groups, but recovered to 63% in convalescent and 64% in the recovered groups, respectively. Serum renin concentrations remain persistently elevated after a episode of takotsubo cardiomyopathy (*P*=0.03 versus controls). Angiotensin converting enzyme levels are significantly depressed during the acute phase compared with convalescent (*P*=0.004), recovered takotsubo cardiomyopathy (*P*=0.02) or controls (*P*=0.03). Angiotensin II is increased in patients with takotsubo cardiomyopathy (*P*<0.001 versus controls) remaining persistently elevated in the chronically recovered group alone (*P*=0.03 versus controls). Big endothelin‐1 levels are unchanged, but endothelin‐1 is significantly lower after takotsubo cardiomyopathy compared with controls (*P*=0.03).

**Conclusions:**

Despite “normalization” of the left ventricular ejection fraction, there is long‐term maladaptive activation of renin‐angiotensin system in patients with takotsubo cardiomyopathy.

**Registration:**

URL: https://www.clinicaltrials.gov; Unique identifiers: NCT02897739, NCT02989454.

Takotsubo cardiomyopathy has become a well‐recognized acute cardiac disease, mimicking an acute myocardial infarction in its presentation, with chest pain/breathlessness, electrocardiographic changes, and biomarker release. The hallmark of the condition is “ballooning” myocardial dysfunction in the absence of culprit lesions on coronary angiography and usually in response to emotional or physical stressful triggers.^1^ Several large registries have reported increased morbidity and mortality of patients with takotsubo cardiomyopathy^2^, ^3^ comparable with that of myocardial infarction but no evidence‐based therapies exist to improve its prognosis, mainly because of a lack of understanding of its pathophysiology. We have recently demonstrated that despite a rapid and spontaneous recovery of the left ventricular ejection fraction, at least a proportion of patients with a previous diagnosis of takotsubo cardiomyopathy evolve towards a heart failure with preserved ejection fraction phenotype, when examined at a median of 2 years post‐acute event.^4^ Neurohumoral activation has been well described in heart failure with reduced ejection fraction, and its antagonism is the basis for modern, first line therapy. In contrast, there is marked heterogeneity in neurohumoral activation in heart failure with preserved ejection fraction,^5^ where neuro‐hormonal blockade trials that collectively included patients of multiple aetiologies were unsuccessful. Here, we investigate if the sequential acute to long‐term response of the renin‐angiotensin and endothelin‐1 systems follows the “normalization” of the left ventricular ejection fraction in patients with takotsubo cardiomyopathy.

## METHODS

### Study Population

Patients with takotsubo cardiomyopathy previously enrolled at the University of Aberdeen/Aberdeen Royal Infirmary between June 2011 to March 2019 (HEROIC [Persistent Symptoms and Early Incomplete Recovery After Acute Stress‐induced Cardiomyopathy: Is There Ongoing Heart Distress? The HEROIC Study] and TERRIFIC [Pathogenesis of Acute Stress Induced (Tako‐tsubo) Cardiomyopathy: Energy Shut‐Down or Intense Inflammation?]) were retrospectively identified based on their clinical characteristics, serum availability in the biobank and medications prescribed. All patients had serial cardiac magnetic resonance imaging scans performed to determine left ventricular ejection fraction. In addition, age‐ and sex‐matched controls were recruited to fit the comorbidity profile of the patients as best possible. The study protocol was reviewed and approved by the institutional review board. All patients gave written informed consent for sampling, storing and subsequent processing of blood. The data that support the findings of this study are available from the corresponding author upon reasonable request.

### Exclusion Criteria

Any patients with endocrine diseases and/or any comorbidities or medications that could influence the biomarkers tested in this report were not selected.

### Blood Sample Timing and Collection

Blood samples were collected during the acute presentation (“acute”), convalescence (3–5 months after presentation, “convalescent”), and at least 1 year after index presentation (“recovered”). In brief, 20 mls blood was sampled in BD Vacutainer SST and EDTA tubes, followed by immediate centrifugation for 15 minutes at 1300 *g*, at room temperature. The resultant serum was carefully pipetted, aliquoted in separate cryovials and immediately stored in a −80 °C biorepository freezer at the University of Aberdeen.

### Measurement of Renin, Angiotensin‐Converting Enzyme and Angiotensin II, Big Endothelin, and Endothelin‐1

Commercially available ELISA kits were used to measure renin (R&D Systems Inc, Human Renin Quantikine ELISA Kit, Abingdon, United Kingdom), angiotensin‐converting enzyme (Abcam, ab263889 Human ACE SimpleStep ELISA Kit, Cambridge, United Kingdom), angiotensin II (Biovision, Angiotensin II (Ang II) (Human) ELISA Kit, Milpitas, Canada), Big Endothelin‐1 (Invitrogen, Human Big ET‐1 Platinum ELISA, Vienna, Austria) and Endothelin‐1 (MyBioSource, Human Endothelin 1 ELISA Kit, California). Between 50 to 100 μL of serum was used for each and the protocol followed the manufacturer's instructions.

### Measurement of B‐Type Natriuretic Peptide

B‐type natriuretic peptide concentration were measured using a benchtop immunoassay (Alere Triage MeterPro); 1 to 2 μL of serum was used for each following the manufacturer's instructions.

### Statistical Analysis

Patients were added to the study based on age and sex to ensure group differences in mean age remained minimal. Data were analyzed by 1‐way ANOVA, and group means were compared by post hoc tests with Tukey adjustment. Some of the outcomes were correlated with age and for these variables we reported the results of an ANOVA in which age was included as a covariate. This analysis was examined for all variables and the significance of age used to assess if the correlation was present. Data are shown as mean±SEMs. Where variable distributions appeared skewed, they were log‐transformed before analysis. It wasn't completely clear from outcome histograms whether a log transform was appropriate or not, so we performed an analysis on both original and log scale. For all results presented, the significance of what is stated was present in both versions. Means are presented from the untransformed version. Calculations were done with R3.6 (R Foundation for Statistical Computing, Vienna). A *P* value <0.05 was considered as statistically significant.

## RESULTS

### Study Patients

Ninety patients with takotsubo cardiomyopathy (n=30 in each of the “acute,” “convalescent,” and “recovered” groups) and 30 controls were studied. Population demographics, including left ventricular ejection fraction at time of acute diagnosis and at time of sampling, and medications are shown in Table 1. Only 1 patient in the “recovered” takotsubo cardiomyopathy group had a small area of fibrosis on cardiac magnetic resonance, presumed to be a previous myocardial infarction which was unknown to the patient. Eighteen patients were part of both acute and convalescent groups.

**Table 1:** Baseline Characteristic in Patients With Takotsubo Cardiomyopathy and Matched Controls

	Takotsubo cardiomyopathy patients			Matched controls	*P* value (ANOVA)
	Acute (n=30)	Convalescent (n=30)	Recovered (n=30)	(n=30)	
Age, y	61 (51–73)	61 (56–70)	66 (58–72)	57 (51–66)	0.406
Female sex, n (%)	30 (100)	29 (97)	30 (100)	28 (93)	0.295
Time to blood sampling from presentation, d	5.65 (1.5–10.5)	154 (112–159)	1419 (747–1904)	N/A	
Left ventricular ejection fraction at presentation (%)	38±1.6	39±2.2	39±4.3	N/A	
Left ventricular ejection fraction at blood sampling (%)	38±1.6	63±2.0	64±2.6	N/A	
Past medical history					
Hypertension, n (%)	0 (0)	0 (0)	0 (0)	0 (0)	
Myocardial infarction, n (%)	0 (0)	0 (0)	1 (3)	0 (0)	0.396
Previous takotsubo cardiomyopathy, n (%)	3 (10)	3 (10)	0 (0)	0 (0)	0.025
Diabetes, n (%)	1 (3)	1 (3)	0 (0)	0 (0)	0.574
Medication history					
Beta‐blocker, n (%)	2 (7)	2 (7)	11 (37)	0 (0)	0.001
Calcium channel blocker, n (%)	0 (0)	0 (0)	0 (0)	0 (0)	
Angiotensin‐converting enzyme inhibitor, n (%)	0 (0)	0 (0)	0 (0)	0 (0)	
Angiotensin receptor blocker, n (%)	0 (0)	0 (0)	0 (0)	0 (0)	
Diuretic, n (%)	1 (3)	1 (3)	7 (23)	0 (0)	0.001
Mineralocorticoid antagonists, n (%)	0 (0)	0 (0)	0 (0)	0 (0)	
Endothelin receptor antagonists, n (%)	0 (0)	0 (0)	0 (0)	0 (0)	
Trigger type					
Emotional, n (%)	15 (50)	16 (54)	15 (50)	N/A	0.842
Physical, n (%)	9 (30)	7 (23)	8 (27)	N/A	0.848
None, n (%)	6 (20)	7 (23)	7 (23)	N/A	0.937
Takotsubo variant					
Apical, n (%)	26 (88)	26 (88)	26 (88)	N/A	
Mid‐cavity, n (%)	3 (9)	3 (9)	4 (12)	N/A	
Basal, n (%)	1 (3)	1 (3)	0 (0)	N/A	
Echocardiographic features					
Left ventricular twist, °	11.66±1.32	14.04±1.83	9.83±5.74	N/A	0.458
Apical circumferential strain, %	−12.14±1.44	−19.64±0.67	−15.06±1.38	N/A	<0.001
Global longitudinal strain, %	−12.13±0.76	−18.52±0.58	−16.31±0.98	N/A	<0.001
Tricuspid regurgitation jet velocity, m/s	2.50±0.13	3.78±1.25	2.27±0.12	N/A	0.461
E/E’ ratio	11.76±0.86	8.8±0.46	7.67±0.82	N/A	0.002

Data are shown as frequencies n (%), ° (degrees of rotation), or mean (interquartile range) or mean±SEM. The ANOVA *P* value reflects significant differences in means across all 4 groups.

### Renin‐Angiotensin Axis in Takotsubo Cardiomyopathy at Presentation and During Follow‐Up

Patients with takotsubo cardiomyopathy had higher renin compared with controls (*P*=0.03; Figure A), and out of the 3 takotsubo cardiomyopathy groups, the renin level in the acute group was significantly elevated compared with healthy controls (*P*=0.03). Angiotensin‐converting enzyme levels were significantly reduced during acute presentation compared with convalescent patients with takotsubo cardiomyopathy (*P*=0.004), recovered takotsubo cardiomyopathy (*P*=0.02) or the control population (*P*=0.03, Figure B). The takotsubo cardiomyopathy population had significantly elevated angiotensin II levels compared with controls (*P*<0.001), and the angiotensin‐II levels were significantly increased in the recovered takotsubo cardiomyopathy group alone compared with the control group (*P*=0.03, Figure C).

**Figure Figure.**
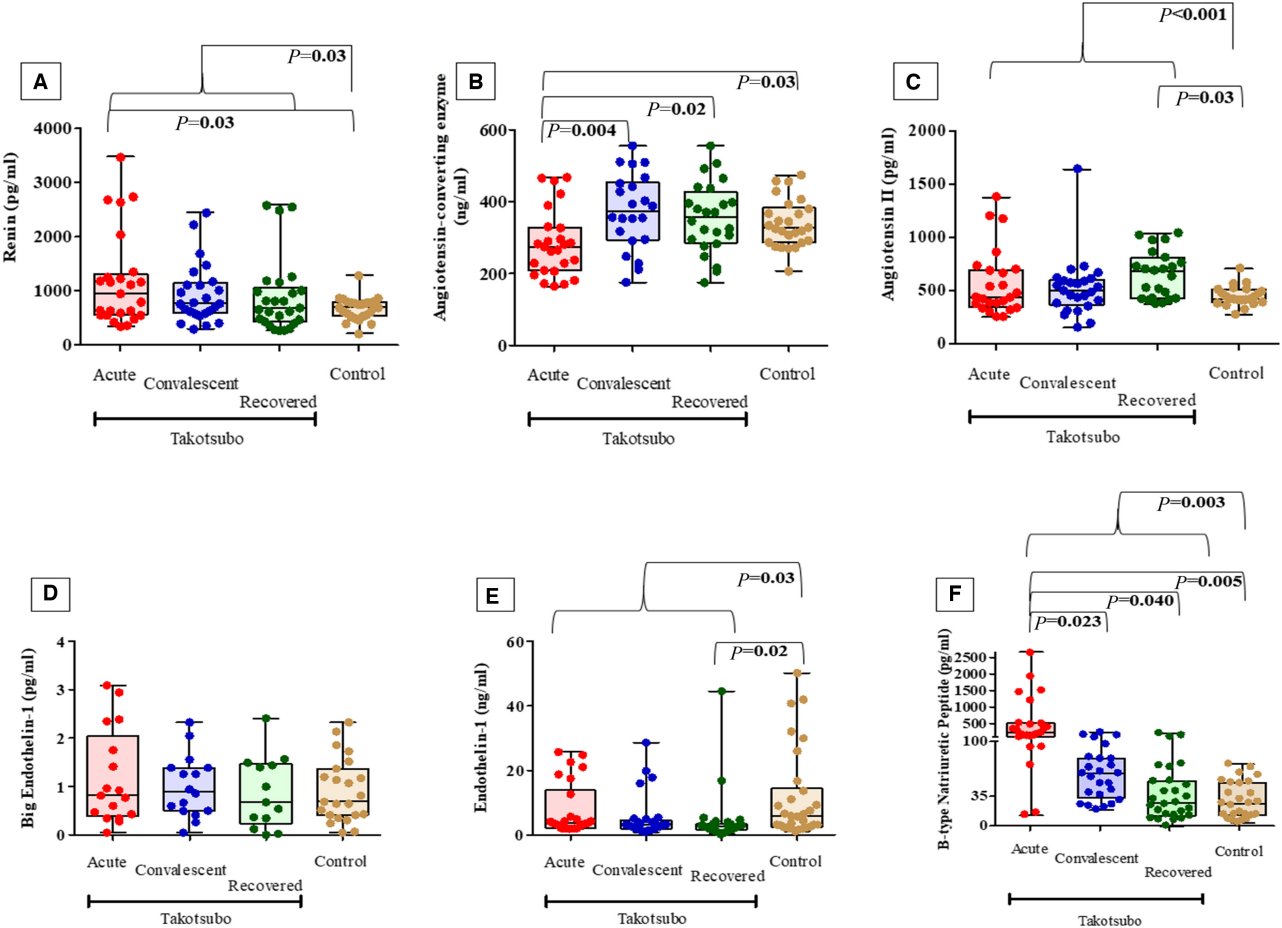
Neurohumoral, endothelin and natriuretic peptide axes in takotsubo cardiomyopathy compared with matched controls. Serum concentrations of renin (**A**), angiotensin‐converting enzyme (**B**), angiotensin II (**C**), big endothelin‐1 (**D**), endothelin‐1 (**E**) and B‐type natriuretic peptide (**F**) in acute (n=30), convalescent (n=30) and chronically recovered (n=30) patients with takotsubo cardiomyopathy and matched controls (n=30). Data presented as median, 25th and 75th centiles, and maximum and minimum (whiskers) with superimposed individual data points.

### Endothelin System in Takotsubo Cardiomyopathy at Presentation and During Follow‐Up

There was no significant difference in big endothelin‐1 serum levels between groups with takotsubo cardiomyopathy during any point in the natural history of the condition or compared with controls (Figure D). The takotsubo cardiomyopathy population had lower endothelin‐1 compared with controls (*P*=0.03; Figure E), with endothelin‐1 level in the chronically recovered takotsubo cardiomyopathy group alone being significantly lower compared with healthy controls (*P*=0.02).

### B‐Type Natriuretic Peptide in Takotsubo Cardiomyopathy at Presentation and During Follow‐Up

The takotsubo cardiomyopathy population had significantly elevated B‐type natriuretic peptide concentration compared with controls (*P*=0.003). B‐type natriuretic peptide concentration was elevated during the acute presentation compared with convalescent takotsubo cardiomyopathy (*P*=0.023), recovered takotsubo cardiomyopathy (*P*=0.04), or control population (*P*=0.005) – Figure F.

All biomarker numerical data and overall ANOVA are presented in Table 2.

**Table 2:** The Renin‐Angiotensin, Endothelin and Natriuretic Peptide Systems in Takotsubo Cardiomyopathy Compared With Matched Controls

	Takotsubo syndrome			Controls	ANOVA *P* value
	Acute (n=30)	Convalescent (n=30)	Recovered (n=30)	(n=30)	
Neurohumoral system					
Renin, pg/mL	1179±173	938±110	884±133	672±41	0.040
Angiotensin converting enzyme, ng/mL	283±19	374±22	355±20	339±13	0.006
Angiotensin‐II, pg/mL	566±63	521±55	651±46	447±19	0.04
Endothelin system					
Big endothelin‐1, pg/mL	1.17±0.24	1.0±0.16	0.88±0.21	0.92±0.14	0.685
Endothelin‐1, ng/mL	7.9±1.6	5.3±1.2	4.5±1.6	11.7±2.5	0.03
Natriuretic peptide system					
B‐type natriuretic peptide, pg/mL	414±123	63±13	43±18	31±4	0.002

Data are shown as mean±SEM. The ANOVA *P* value reflects significant differences in means across all 4 groups.

## DISCUSSION

We demonstrate major acute (adaptive) and chronic (maladaptive) activation of the neurohumoral system and a persistent reduction in the biologically active endothelin‐1 in patients with takotsubo cardiomyopathy.

During any acute heart failure episode or further decompensation, the renin‐angiotensin system becomes activated resulting in elevated plasma neurohormone concentrations. In the subsequent chronic stages of “stable” heart failure, serum neurohormone concentrations decrease but do not normalize. On the other hand, endothelin‐1 and its precursor, big endothelin‐1, are increased throughout. We selected a representative cohort of patients with takotsubo cardiomyopathy with no confounding comorbidities or medications that could affect the renin‐angiotensin or endothelin systems, to investigate if the renin‐angiotensin and endothelin‐1 response pathways follow the same pattern of recovery as the left ventricular ejection fraction.

The present study demonstrates a similar dysregulation of the renin‐angiotensin system as seen in classical heart failure syndromes of other etiologies, with increases in renin and, in the longer term, angiotensin II concentrations.^6^ We did observe an initial fall in angiotensin‐converting enzyme concentrations that may reflect compensatory, alternative pathways of angiotensin II synthesis regulated by the significantly elevated natriuretic peptides.^7^, ^8^ However, while such regulatory mechanisms of angiotensin II synthesis may be required to maintain hemodynamic stability in the acute setting and are most likely adaptive, the significantly increased angiotensin II concentration in the chronically recovered patients imply a sustained, maladaptive neurohormonal activation. Such chronic increase in angiotensin II mediates profibrotic and proinflammatory pathways in the myocardium, which are in keeping with the concept of heart failure with preserved ejection fraction previously demonstrated by our group in patients post‐takotsubo cardiomyopathy.^4^, ^9^ The predominant echocardiography findings in patients with takotsubo cardiomyopathy are decreased global longitudinal strain, apical circumferential strain, and left ventricular twist and torsion.^4^, ^10^ Global longitudinal strain and apical circumferential strain improve during the convalescent period (median 5 months) but worsen during the recovered period (median 47 months) matching the trend seen with angiotensin II levels. Despite their persistently elevated B‐type natriuretic peptide levels, patients with takotsubo cardiomyopathy have normal estimated left ventricular filling pressures and normal estimated pulmonary artery pressures at rest (Table 1). It is possible that these become elevated with exercise, as is often the case in patients with heart failure with preserved ejection fraction.^11^

Therefore, our findings demonstrate for the first time that patients with takotsubo cardiomyopathy have similar maladaptive mechanisms of renin‐angiotensin system activation as in typical heart failure syndromes with reduced ejection fraction, suggesting that these abnormalities could be targets for angiotensin‐converting enzyme inhibition. Coupled with registry data which point to a significant survival benefit of this class of drugs,^2^ it follows that angiotensin‐converting enzyme inhibition may have therapeutic potential and would be prime candidate for randomized controlled trials of improving the long‐term outcomes for patients with takotsubo cardiomyopathy.

We demonstrate sustained downregulation of endothelin‐1 despite normal big endothelin‐1 concentrations in patients with takotsubo cardiomyopathy. Endothelin‐1 is known to be increased in other heart failure syndromes, acute myocardial infarction as well as populations with high degree of comorbidities such as hypertension and diabetes.^12^ Interestingly, 1 previous publication reported increased plasma endothelin‐1 concentrations in takotsubo cardiomyopathy,^13^ however half of their cohort had comorbidities such as hypertension, diabetes, hyperlipidemia, or were smokers and they were significantly medicated (a third were on angiotensin‐converting enzyme and angiotensin II blockers and a third on beta‐blockers) – in contrast to our takotsubo cardiomyopathy cohort who were free of any comorbidities and on no medication. It is therefore possible that the elevated endothein‐1 concentrations may have been confounded by patients’ comorbidities and/or medications in previous report.^13^

In our study we observe the opposite to Jaguszewski et al, specifically decreased endothelin‐1 concentrations. This can occur in 1 of 2 ways: (1) Reduced activity of the endothelin converting enzyme or (2) Enhanced endothelin‐1 clearance.^14^, ^15^ Endothelin‐1 clearance is mediated by the endothelin type B receptor and blockade of this receptor causes increased plasma endothelin‐1 concentrations without affecting plasma big endothelin‐1 concentrations. Endothelin type B receptor mediates vascular smooth muscle vasoconstriction, suggesting an enhanced endothelin‐1 mediated vasomotor tone in patients with takotsubo cardiomyopathy. It is possible that the decrease in endothelin‐1 seen in takotsubo cardiomyopathy is because of increased activity/density of endothelin type B receptors with vascular smooth muscle tone changes and a reduction in serum concentration of endothelin‐1, without affecting big‐endothelin‐1 levels. Dysregulation of endothelin system and its influence on vasomotor tone is worthy of further exploration in takotsubo cardiomyopathy.

## CONCLUSIONS

Overall, our findings show that despite “normalization” of the left ventricular ejection fraction, there is long‐term maladaptive activation of renin‐angiotensin system and dysregulation of endothelin‐1 in patients with takotsubo cardiomyopathy. This has implications for potential therapeutic interventions in exploring if reduction in angiotensin II levels result in improved long‐term outcomes after takotsubo cardiomyopathy.

## Sources of Funding

This work was supported by British Heart Foundation PG/15/108/31928, FS/RTF/20/30009, the 2016 Josephine Lansdell BMA Award, Chief Scientist Office Scotland CGA‐16‐4 and NHS Grampian Endowment ES868.

## Disclosures

None.

## References

[1] Sharkey SW , Windenburg DC , Lesser JR , Maron MS , Hauser RG , Lesser JN , Haas TS , Hodges JS , Maron BJ . Natural history and expansive clinical profile of stress (tako‐tsubo) cardiomyopathy. J Am Coll Cardiol. 2010;55:333–341. doi: 10.1016/j.jacc.2009.08.057 20117439

[2] Templin C , Ghadri JR , Diekmann J , Napp LC , Bataiosu DR , Jaguszewski M , Cammann VL , Sarcon A , Geyer V , Neumann CA , et al. Clinical features and outcomes of takotsubo (stress) cardiomyopathy. N Engl J Med. 2015;373:929–938. doi: 10.1056/NEJMoa1406761 26332547

[3] Redfors B , Jha S , Thorleifsson S , Jernberg T , Angerås O , Frobert O , Petursson P , Tornvall P , Sarno G , Ekenbäck C , et al. Short‐ and long‐term clinical outcomes for patients with takotsubo syndrome and patients with myocardial infarction: a report from the Swedish coronary angiography and angioplasty registry. J Am Heart Assoc. 2021;10:e017290. doi: 10.1161/JAHA.119.017290 34465127 PMC8649294

[4] Scally C , Rudd A , Mezincescu A , Wilson H , Srivanasan J , Horgan G , Broadhurst P , Newby DE , Henning A , Dawson DK . Persistent long‐term structural, functional, and metabolic changes after stress‐induced (takotsubo) cardiomyopathy. Circulation. 2018;137:1039–1048. doi: 10.1161/CIRCULATIONAHA.117.031841 29128863 PMC5841855

[5] Vergaro G , Aimo A , Prontera C , Ghionzoli N , Arzilli C , Zyw L , Taddei C , Gabutti A , Poletti R , Giannoni A , et al. Sympathetic and renin‐angiotensin‐aldosterone system activation in heart failure with preserved, mid‐range and reduced ejection fraction. Int J Cardiol. 2019;296:91–97. doi: 10.1016/j.ijcard.2019.08.040 31443984

[6] Paz Ocaranza M , Riquelme JA , García L , Jalil JE , Chiong M , Santos RAS , Lavandero S . Counter‐regulatory renin–angiotensin system in cardiovascular disease. Nat Rev Cardiol. 2020;17:116–129. doi: 10.1038/s41569-019-0244-8 31427727 PMC7097090

[7] George J , Mackle G , Manoharan A , Khan F , Struthers AD . High BNP levels in rheumatoid arthritis are related to inflammation but not to left ventricular abnormalities: a prospective case‐control study. Int J Cardiol. 2014;172:e116–e118. doi: 10.1016/j.ijcard.2013.12.119 24433615

[8] McFarlane SI , Winer N , Sowers JR . Role of the natriuretic peptide system in cardiorenal protection. Arch Intern Med. 2003;163:2696–2704. doi: 10.1001/archinte.163.22.2696 14662623

[9] Jugdutt BI . Apoptosis after reperfused myocardial infarction: role of angiotensin II. Experimental and clinical cardiology. 2004;9:219–228. 19641712 PMC2716282

[10] Schwarz K , Ahearn TS , Srinivasan J , Neil CJ , Rudd A , Jagpal B , Frenneaux MP , Pislaru C , Horowitz JD , Dawson DK . Alterations in cardiac deformation, timing of contraction and relaxation and early myocardial fibrosis accompany the apparent recovery of acute stress‐induced (takotsubo) cardiomyopathy: an end to the concept of transience. Journal Am Soc Echo. 2017;30:745–755. doi: 10.1016/j.echo.2017.03.016 28599831

[11] Borlaug BA , Blair J , Bergmann MW , Bugger H , Burkhoff D , Bruch L , Celermajer DS , Claggett B , Cleland JGF , Cutlip DE , et al. Latent pulmonary vascular disease may Alter the response to therapeutic atrial shunt device in heart failure. Circulation. 2022;145:1592–1604. doi: 10.1161/CIRCULATIONAHA.122.059486 35354306 PMC9133195

[12] Miyauchi T , Yanagisawa M , Tomizawa T , Sugishita Y , Suzuki N , Fujino M , Ajisaka R , Goto K , Masaki T . Increased plasma concentrations of endothelin‐1 and big endothelin‐1 in acute myocardial infarction. Lancet. 1989;2:53–54. doi: 10.1016/S0140-6736(89)90303-6 2567834

[13] Jaguszewski M , Osipova J , Ghadri JR , Napp LC , Widera C , Franke J , Fijalkowski M , Nowak R , Fijalkowska M , Volkmann I , et al. A signature of circulating microRNAs differentiates takotsubo cardiomyopathy from acute myocardial infarction. Eur Heart J. 2014;35:999–1006. doi: 10.1093/eurheartj/eht392 24046434 PMC3985061

[14] Plumpton C , Ferro CJ , Haynes WG , Webb DJ , Davenport AP . The increase in human plasma immunoreactive endothelin but not big endothelin‐1 or its C‐terminal fragment induced by systemic administration of the endothelin antagonist TAK‐044. Br J Pharmacol. 1996;119:311–314. doi: 10.1111/j.1476-5381.1996.tb15987.x 8886414 PMC1915875

[15] Goddard J , Johnston NR , Cumming AD , Webb DJ . Fractional urinary excretion of endothelin‐1 is reduced by acute ETB receptor blockade. Am J Physiol Renal Physiol. 2007;293:F1433–F1438. doi: 10.1152/ajprenal.00101.2007 17855483

